# Lack of family education in boarding primary schools in China's minority areas: A case study of Stone Moon Primary School, Nujiang Lisu Autonomous Prefecture

**DOI:** 10.3389/fpsyg.2022.985777

**Published:** 2023-01-05

**Authors:** Guo Guo, Yao Chen

**Affiliations:** ^1^Faculty of Education, Yunnan Normal University, Kunming, China; ^2^Faculty of Teacher Education, Kunming University, Kunming, China

**Keywords:** ethnic areas, boarding primary schools, the transfer of Space-Time, China's minority areas, lack of family education

## Abstract

The lack of family education in boarding primary schools in ethnic minority areas negatively impacts students' physical and mental health. This study constructed an analytical framework for the lack of family education in boarding primary schools from the perspective of Overlapping Spheres of Influence theory. Stone Moon Primary School of Lisu in Nujiang Prefecture was used as the field site for questionnaire survey. The correlation analysis showed that “teachers' avoidance of educational risks”, “the priority in the schooling system of classic hard skills over soft skills” and “the capacity limit of policies and regulations” had significant positive correlation with the degree of “the lack of family education”. In the regression analysis, the adjusted R^2^ was 0.607, indicating that the proportion of “lack of family education” explained by the three factors of “teachers' avoidance of educational risks”, “the priority in the schooling system of classic hard skills over soft skills” and “the capacity limit of policies and regulations” was 60.7% through the regression relationship. Possible solutions include establishing a responsibility standard system and accountability system between teachers and parents, and strengthening the compensation management of family education function in boarding primary schools in ethnic minority areas.

## 1. Introduction

### 1.1. Literature review and research problem

The remote geographical location of minority areas, inconvenient travel logistics, and scattered population result in the proliferation of boarding schools. Boarding school life without family care has serious psychological effects on young students. Academic relevant studies on the lack of family education in boarding schools are mainly divided into four aspects. The first aspect is identity. French Professor Behaghel et al. stated that boarding schools provide opportunities for upper-class students to train their future social competitiveness and they reduce the sense of happiness among students from disadvantaged families (Behaghel et al., 2017). Dr. Jim Cummins from the University of Toronto summarized three distinctive components that were crucial to successful schools: affirmation of students' cultural identity and encouragement of native language literacy; encouragement of active parental participation; and cognitively challenging instruction that provides students to draw on their background experiences while exploring issues that are relevant to their lives. These three distinctive components show the centrality of identity issues in students' successful achievements in school (Cummins, 2001). The second aspect is language. Dr. Jim Cummins put forward the Model of Bilingual Education Interdependence theory, that is, the degree of development of the first language will have a great impact on the acquisition of the second language (Cummins, 2001). Bahry (2009) from the University of Toronto found that the students in the minority areas of Gansu province are often silent for 1–2 years at the beginning of schooling until they have learned Mandarin well enough to understand lessons and express themselves. Stephen May from the University of Auckland found that children immersed in such an absence of familiar environment after 1–2 years of silence can develop basic interpersonal communication skills in the second language, but may lag behind mainstream language learning peers in the second language vocabulary development for 5–7 years as their cognitive academic language development has to catch up to that of native speakers (May, 2016). The third aspect is culture. Chinese scholar Qian Minhui studied the phenomenon of minority students running away and dropping out from consolidated boarding schools in Gansu, interviewing dropouts from three nationalities. The results of the analysis showed that the dropout rate of the students who have a religious belief is higher than those who have none. Students who are able to return home every evening have a much lower dropout rate than boarders. The author explained this phenomenon using Bourdieu's concept of cultural capital: the fact that what constitutes cultural capital at home is not consistent with what constitutes cultural capital at school leads to academic failure and dropout (Qian, 2007). The fourth aspect is psychology. Professor Renfu Luo et al. of Peking University found that poor services in boarding schools and inadequate nutrition intake are the main causes of low student height-for-age Z-scores (HAZ). Therefore, one can infer that malnutrition leads to physical retardation, which may affect the individual's cognitive and emotional development (Luo et al., 2009). In a study of 115 students in a boarding school, Professor Shirley Fisher et al. from University of Dundee found that more than 71% of the group reported having experienced homesickness during the school year. This same group is also reported a higher incidence of non-traumatic ailments during the year and more days off school (Fisher et al., 1986). British scholar Joy Schaverien believed that boarding schools have caused mental trauma among students (Schaverien, 2004). In the follow-up study of boarding students in Australia, Dr. Mander et al. from the University of Western Australia found that students with primary school boarding experience have a significantly higher incidence of mood disorders than non-boarding students after entering secondary school, and are more likely to have negative emotions such as emotional distress, depression, and anxiety (Mander et al., 2015). Research showing the positive effects of boarding also emerged. Martha Vicinus, a professor from the University of Michigan, United States, believed that boarding life positively influences the emotional development of female students (Vicinus, 1984). Reuven Kahane, a scholar at the Hebrew University of Jerusalem, Israel, believed that boarding schools provide opportunities for students to experience roles and rules, thus promoting students' all-round development (Kahane, 1988). Professor Andrew J. Martin et al. from the University of New South Wales found that boarders are able to obtain the professional educators' education on a more continuous basis than non-boarders (Martin et al., 2014). Compared with college students, boarding life leaves a stronger negative impact on middle-school students. By interviewing 447 Indians in Alaska, USA, Professor Evans-Campbell Teresa et al. from the University of Washington found that those with boarding experience in middle school are more likely to suffer from anxiety disorder, post-traumatic stress disorder, and suicidal thoughts than those without boarding experience (Evans-Campbell et al., 2012). Based on a study of 2,953 people in Canada, Professor Elias Brenda from the University of Manitoba demonstrated that the boarding experience of Indian middle schools may lead to later vicious behaviors such as abuse and suicide, and other malignant behaviors and that the potential effects can be profound, even transmitted across generations (Brenda et al., 2012). Professor Md. Alamgir Kabir of Bangladesh and his team analyzed the results of the *t*-test (*p* < 0.001) and found that the average score between residential and non-residential students at the University of Dhaka is significant. Research showed that residential students feel more stressed, lonely, and depressed than non-residential students (Kabir et al., 2018).

Previous studies mostly focus on the boarding life of middle-school and college students. This study focuses on boarding primary schools in ethnic areas because pupils' self-care ability is relatively weak, and the emotional dependence on their parents is stronger. Poor economy and infrastructure of ethnic minority areas make local boarding school conditions more difficult. The multiple circumstances often leave parents with no better choice than sending their children to boarding school. Stone Moon Primary School embodies all the characteristics of boarding primary schools in ethnic areas of China: remote geographical location, inhabitation by ethnic minorities, poor condition of family, weak awareness of family education, younger boarding students, and establishment for the achievement of educational equity. The importance of family education has been repeatedly demonstrated by education experts (e.g., Leichter, 1974; Barrera and Li, 1996; Miu, 2009; Darling et al., 2022). Pupils' opportunities to enjoy family education in ethnic areas have been reduced or even lost since boarding. It may be possible to create a campus environment of family compensation, which can compensate for the lack of family affection among students in boarding schools. Therefore, this study is representative of certain significance. Based on the above findings, it is of practical significance to study the impact of the lack of family education in boarding primary schools in ethnic areas and the possible solutions to such problems. From the psychological or emotional effect on language minority children of boarding schools, we can see the separation of the care and the love of family further exacerbated by removal from a familiar cultural and linguistic environment. The challenge faced by boarding primary schools in ethnic areas is twofold: 1. the absence of familiar culture and language in school lessons in Mandarin-dominant curriculum and pedagogy; and 2. the absence of contact with family, home culture and language outside of lessons as minority children live in dormitories. The two challenges are related to reducing the quality of learning of curriculum contents without guaranteeing advanced learning of the second language and creating a feeling of inferiority with regard to minority identity, culture, and language. Especially for first graders, as they do not understand the lessons and what is going on around them and cannot express themselves. For the all-round and harmonious development of students, boarding schools should face the challenges and undertake the obligation to offset the lack of family education. Paul Durning, an educator, put forward the concept of “family-oriented parenting” in the mid-1980s (Fablet, 2005). He interpreted the “family-oriented parenting” as “agency” rather than “substitution” (Wu and Hervé, 2018), that is, social care and education institutions, as the agents of parents and families, undertake the care and education of children in a certain period of time. Drawing on the concept of “family-oriented parenting,” this study refers to the education implemented by boarding schools to offset the lack of family education as “family-oriented education.” This family-oriented education management mode is a process of combining quality education with life education, aiming at students' development, according to students' growth needs and the specific lifestyle of different families. On the basis of the above, this study investigates the actual performance of the lack of family education in boarding primary schools in ethnic areas, putting forward the hypothesis of the causes of it from the perspective of Overlapping Spheres of Influence, using empirical research to verify the hypothesis, and exploring ways to solve this difficult problem.

### 1.2. Field introduction

Nujiang Lisu Autonomous Prefecture is located in the northwest of Yunnan Province, China. It is the junction of China and Burma. It covers an area of 14,703 square kilometers. The minority population of Nujiang Prefecture accounts for 92.2%, among which Lisu accounts for 51.6%. According to the China Statistical Yearbook (National Bureau of Statistics., 2021), the population of Nujiang Lisu Autonomous Prefecture is 552,694.

Nujiang Prefecture includes Lushui City, Fugong County, Gongshan Dulong and Nu Autonomous County, Lanping Bai and Pumi Autonomous County. Nujiang Prefecture is the only Lisu Autonomous Prefecture in China, among which Dulong and Nu are the unique minorities in Nujiang. Nujiang Prefecture is the autonomous prefecture with the largest ethnic composition and the largest minority population in China (Nujiang Lisu Autonomous Prefecture., 2022).

The people of Lisu are from a South-Asian type of Mongolian race. Its national language belongs to the Tibeto-Burman Language, Yi, which is a branch of the Sino-Tibetan language family. Lisu people have their own religion. Lisu ethnic groups originated from the ancient Diqiang ethnic group and are related to Yi. The Lisu ethnic group is primarily distributed along the Nu River and Enmei Kai River (tributaries of Irrawaddy River) areas, and the rest are scattered in other areas of Yunnan, Northeast India, and the border areas of Thailand and Myanmar (Ethnic Lisu., 2022).

**Figure 1:**
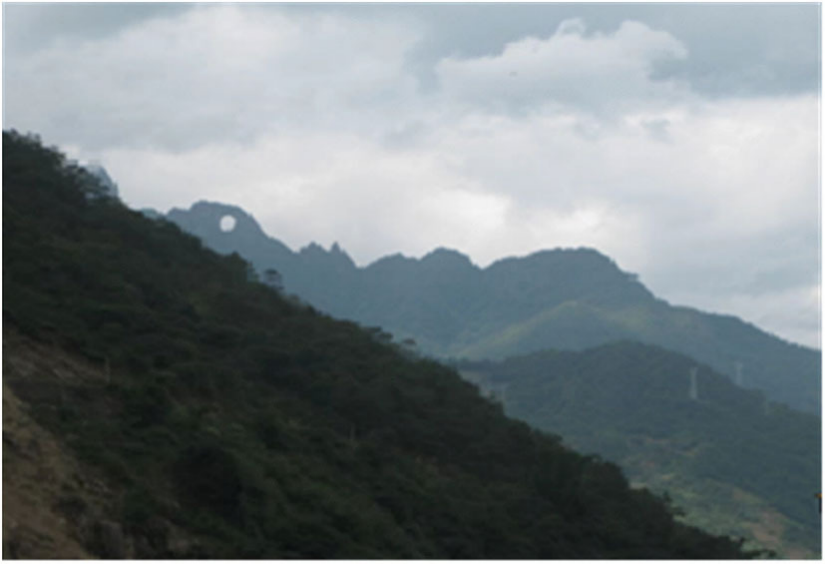
Stone Moon.^1^

**Figure 2:**
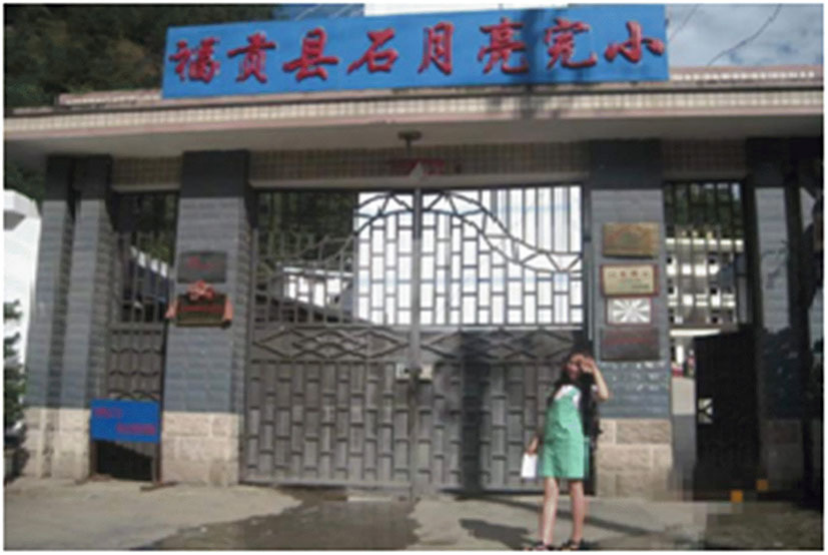
Stone Moon Primary School.^2^

**Figure 3:**
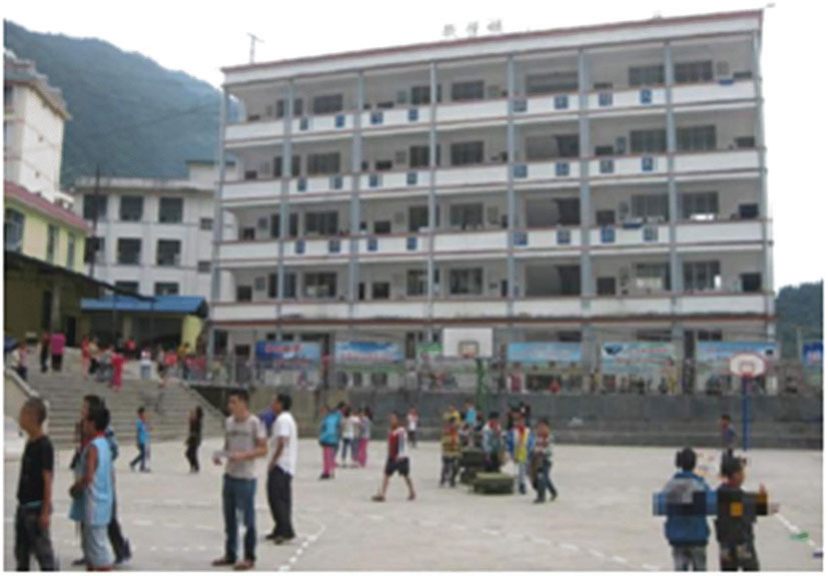
The campus of Stone Moon Primary School.

At the peak of Gao-Ligong Mountain, there is a huge marble cave, which is 40 meters wide, 60 meters high, and 100 meters deep. It has been given the beautiful name “Stone Moon” (Stone-Moon., 2022) (Figure 1). Stone Moon Primary School is located on Lishadi Street, Stone Moon Township, 42 kilometers north of Fugong County (Figures 2, 3). The school was founded in 1922 and moved to the present site in 1979 due to geological disasters. In September 2007, it became a nine-year system school. In March 2011, it was split into two independent schools, namely, Stone Moon Primary School and Stone Moon Middle School. At present, the school serves a radius of 8 km, covering 57 villager groups of five villager committees, with a population of 8,256. The school covers an area of 9,444.75 square meters (14.03 square meters per student), a campus green area of 1,987 square meters (2.95 square meters per student), a construction area of 5,478 square meters (8.14 square meters per student), and a sports field of 4,613 square meters (6.85 square meters per student). The main buildings of the campus are the teaching buildings, student dining halls, student dormitories, office buildings, toilets and bathrooms, and teacher accommodation buildings. There are 15 ordinary classrooms, 12 functional rooms, and 57 student dormitories. The school layout is reasonable and the education and teaching facilities are complete. There are two laboratories and two instrument rooms, with teaching instruments and equipment according to the first-class standard. There is one computer room, one library, and seven classrooms for interest groups, with all the audio, physical, and aesthetic equipment matching first-class standards. At present, there are 15 compulsory classes with 673 students, among which 660 are of the Lisu ethnic group, accounting for 98.06% of the total number of students in the school. Of the remaining, 13 are of the Nu ethnic group, accounting for 2% of the total number of students in the school, and one student is of Han ethnicity. This student enjoys the same educational treatment as local students, including exemptions of text book fees, miscellaneous fees, and accommodation fees. The enrollment rate of school-age children in the area has reached 100%, and the retention rate of students in recent years is above 99.5%. There are seven kindergartens with a total of 272 children in the school, with 45 faculties and staff members, including 45 full-time teachers, 25 bachelor's degree teachers, and 15 junior college degree teachers. The ratio of students to faculty is 15:1. There are 20 preschool volunteer teachers, 2 temporary teachers, 11 temporary workers, and 2 security guards. At present, the campus broadband network has been opened, a campus website has been built, and the construction of a modern education information network, innovative teaching, and scientific research work is steadily advancing (Nu, 2020).

## 2. Theory and hypothesis

### 2.1. Reality of the lack of family education in boarding primary schools in ethnic areas

The lack of family education in boarding primary schools in ethnic areas is mainly reflected in three aspects: first, the lack of family education contents; second, the absence of family education emotion; and third, the Space-Time migration of family education.

#### 2.1.1. Lack of family education contents

Family education is a kind of education peculiar to human beings, which is mainly reflected in the educational influence of parents on children, and also includes the interaction of communication and behavior among members. Family education in a broad sense includes parents' educating their children, children's influence on parents, and even the educational influence between parents, children, and grandparents. In a narrow sense, family education mainly refers to the educational influence of parents on their children (Miu, 2009). The mannerisms of family members directly reflect the family ethos. The President of the People's Republic of China Xi Jinping put forward that it was necessary to build a family civilization from three aspects: family tradition, family discipline, and family construction (Xi, 2017). For individuals, the function of the family is to provide basic human needs, enhance the individual's sense of belonging, and relieve people's tension and stress. For society as a whole, the function of the family is the reproduction of people, the cultivation of human socialization, and the education of individual social norms and social values (Eshleman, 1974). Parsons and Bales argued that the basic function of the family was bearing children, cultivating people's primary socialization, and promoting the stability of adult personality (Parsons and Bales, 1955). People who are satisfied with family life, are mentally relaxed, have a sense of belonging and emotional stability, and abide by rules, can promote the construction of family civilization, better integrate into society, and create value for society. Because the construction of family civilization is an important base point for economic development and social harmony. Family education is the fundamental path to promote the construction of family civilization. Therefore, the contents of family education should also be developed around the above three aspects.

Family tradition has multiple pillars. Without the cooperation of parents, schools cannot perform their educational functions. In order to protect the transmission of moral values from generation to generation, the inheritance function of family traditional culture is irreplaceable (Birquier et al., 1998a). For this reason, the primary pillar of family tradition should be teaching individuals ways to enhance self-cultivation and shape moral behavior. The form of the family, like the form of social organization, is both a product of the kinship system and a combination of constraints that reflect the social system in which the family lives (Birquier et al., 1998b). Therefore, the second pillar of family tradition is the idea of regulating and restricting the behavior of family members and promoting the construction and development of the family. Family can be regarded as a social group or social organization to regulate behavior and etiquette (Eshleman, 1974), and the behavior and etiquette of individuals in the family will affect the whole society to a certain extent. At this level, the behavior and etiquette that family members display to the outside world is an extension of the family members in social relations and is also the third pillar of building and keeping family traditions. In all societies, the family is the premier institution for all of the following: socialization of children, adult intimate relationships, life-long economic support and cooperation, and continuity of relationships along the life-course (Hammond, 2010). The spirit of innovation is the condition to promote the socialized development of children, rigorous work is the basis to improve the family's economic condition, and harmonious interpersonal relationship is the guarantee of smooth cooperation. As an abstract concept, the influence of family tradition on people can be reflected in practical actions. Cultivating an individual's pursuit of learning innovation, rigorous work, and interpersonal harmony is the fourth pillar of family tradition. Goode argues that the development of the family does not depend entirely on the political and economic structure of the country, and in fact, they are interdependent (Goode, 1982). Thus, the development of the country also depends on the attitudes and actions of specific family members to protect the country. Cultivating and guiding individuals to be patriotic and serve the family and country is the fifth content of building and keeping family traditions (e.g., Li, 2020; Zeng, 2020). According to the investigation, Stone Moon Primary School has not set up courses for family tradition education. To some extent, boarding schools delay the students' inheritance of family traditions.

The contents of family discipline depend on the purpose of family education. Good family discipline is the premise and foundation of the healthy development of teenagers and children, and it has a profound influence on the happiness of families and the stability and progress of society. Therefore, the specific contents of family discipline should include all knowledge and skills that can promote family happiness, children's development, and social progress. Chinese educator Chen Heqin mentioned that moral education, aesthetic education, intellectual education, physical education, and labor education should form parts of the basic contents of family discipline (Chen, 1981).

According to the requirements of the Chinese government, the Stone Moon Primary School offers one moral education lesson, one art lesson, one physical education lesson, one music lesson, and one labor education lesson every week. There are Chinese and math curriculums every day. Occasionally, the art and physical education classes are occupied by teachers who teach other subjects. Moral education and labor education have relatively high requirements for teachers' demonstration and students' practicality. That is to say, such courses require students to be enlightened in life practice, and at the same time, they also need the teachers' personal demonstration. Therefore, even if boarding schools that are separated from family education offered these two courses, the results would not be satisfactory. Action depends on cognition, so the cognition “paying attention to the family” is the premise of family construction. In essence, family is one of the earlier and more important social relationships. The family is the basic cell of society in essence, and family education is the basic point of education. “Family-oriented” should be committed to realizing the dream and happiness of every member of the family. The contents of family construction should include respect for every family member, building a harmonious family atmosphere, and striving to improve the value of family happiness.

Stone Moon Primary School does not offer a relevant course on family construction. Teachers and students are also unaware of the significance of family construction for education. This is not an isolated situation, and most schools, including universities, do not offer courses on family construction. Family construction is the basic content of family education, and family education is the basic form of education. Education is the primary productive force. Therefore, the lack of family construction content in boarding schools in ethnic areas is an invisible incentive for the intergenerational transmission of poverty. It is not only about the perpetuation of material poverty, but also about the transmission of emotional poverty. The most precious things in the world tend to be free, such as sunshine, air, companionship, and love. All these require our sincere empathy and emotional experience. Emotional poverty is the real poverty.

#### 2.1.2. Absence of family emotional education

Family emotional education refers to parents setting up family education, paying attention to children's emotional experiences, and ensuring that children have positive emotional experiences to achieve spiritual pleasure and inner prosperity. There are three levels of family emotional education: first, parents provide material and spiritual care to their children; second, parents guide their children to learn emotional experience. third, parents guide their children to improve their emotional value identification. In traditional Chinese culture, “Qin” mainly refers to the father and mother, namely parents, and is extended to relatives within the family; “Qing” means affection. Family affection is the general term of affection between family members, including affection between parents and children and affection between brothers and sisters, among which affection between parents and children is the main natural basis of family affection (Ye, 2011). Family affection is critical for growing children and is the greatest spiritual wealth. Children aged 6–12 came to Stone Moon Primary School with family warmth stripped and family warmth blocked and were unable to feel the material and spiritual care from their parents. Due to the lack of parental dependence and care, boarders lose emotional support. The friendship between boarders and the care of teachers is insufficient to replace their parents in the short term. The lack of emotional security makes them insecure (Dong, 2012), leaving deep scars in their hearts. As time goes by, these long-term boarding students will lose their strong relationship with their parents, and some will often feel lonely and helpless. They may gradually shut down and be overcome by an inferiority complex, and even produce a series of serious psychological problems.

Students at Stone Moon Primary School return home on the 8th, 18th, and 28th of every month. Holidays end on the 10th, 20th, and 30th^3^ of every month and students return to school. The students spend only 6 days per month at home. Within the family, parents should guide their children's emotional experience in the aspects of survival experience (labor education), cognitive experience (cognitive of the family), self-experience (self-knowledge and acceptance), communication experience (interaction between family members), aesthetic experience (truth, goodness, and beauty of the family), moral experience (family ethics), and sane experience (the emotions that arise in the process of understanding and evaluating the family). The emotional experience that boarding primary school students can feel during such a short vacation is only the “sadness of parting”. There are no specific family experience courses at Stone Moon Primary School. The atmosphere that students feel at school primarily consists of school education, which reinforces their yearning for their parents.

Family emotional value identification refers to parents paying attention to their children's emotions and emotional values, guiding them to fully understand the value and significance of emotions, improving their psychological ability to resist pressure, and helping them love life, be grateful for life, respect nature, and appreciate life. In the family, group meals and bedtime conversations are golden moments for family members to warm their hearts and support each other spiritually. The identification of family emotional value is most easily generated in these two links. Many studies have pointed out that family leisure activities are important elements in developing a close and healthy relationship between couples and their children, enhancing family emotional value identification and promoting family function (Chen, 1999; Liu, 2011). Studies have consistently shown that family leisure activities are positively correlated with family life satisfaction and family emotional connection (Holman and Epperson, 1984; Holman and Jacquart, 1988; Hawkes, 1991).

**Figure 4:**
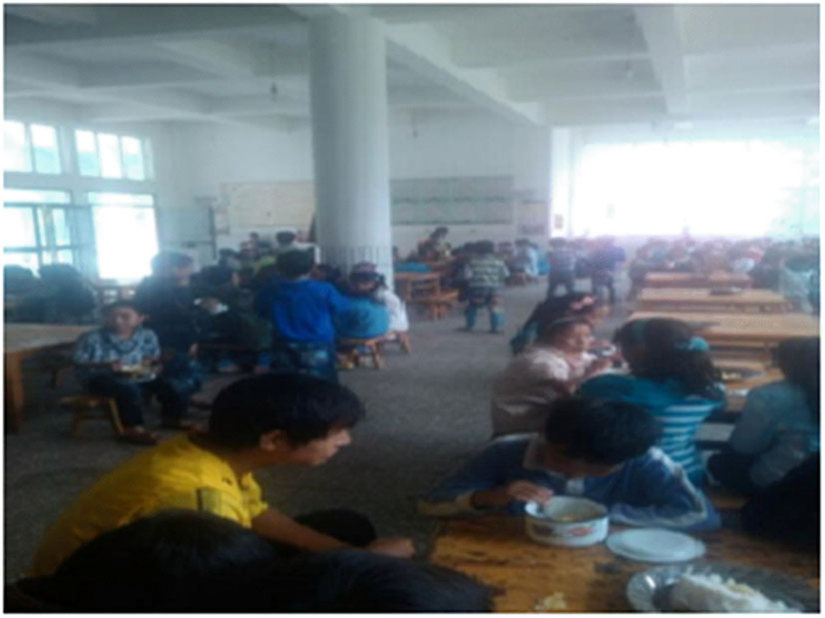
Students at dinner time.

**Figure 5:**
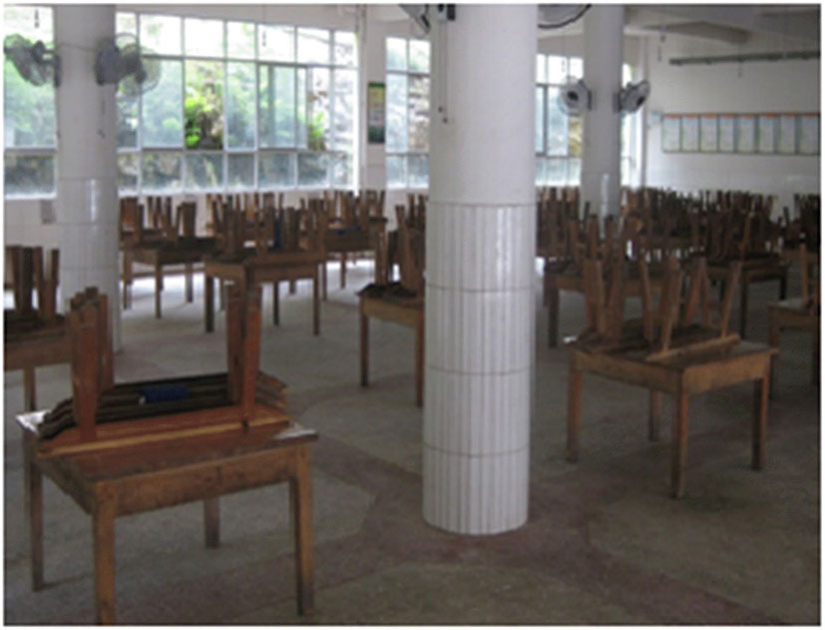
Dining room.

**Figure 6:**
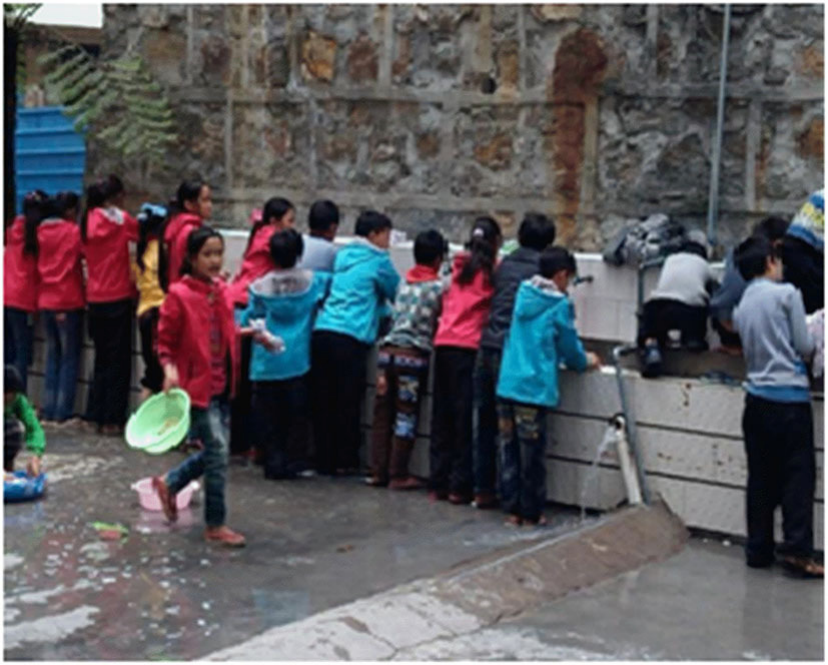
Sleeping preparation.

**Figure 7:**
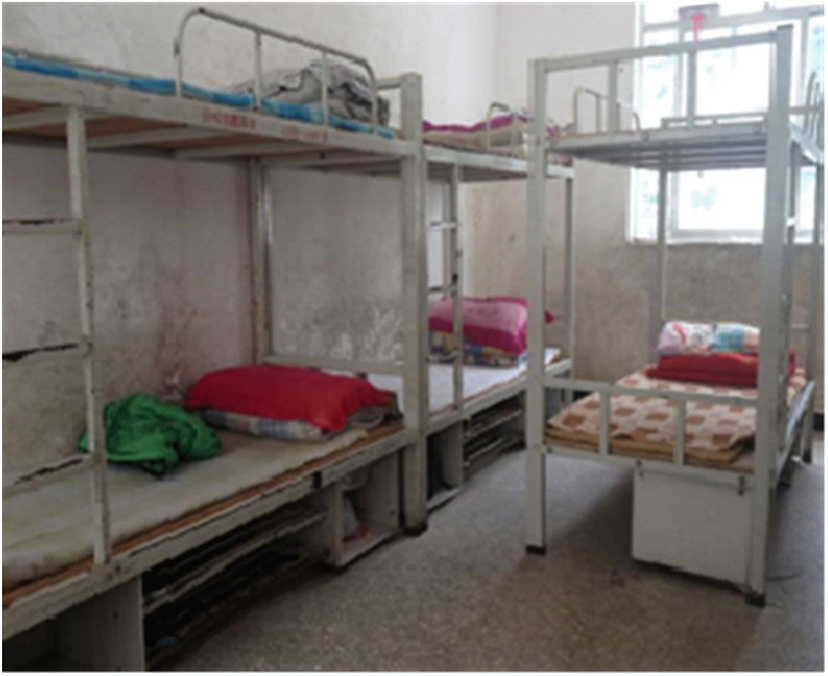
Dormitory arrangement.

However, the boarding system of the Stone Moon Primary School embodies the binding of school management, and makes the students “quick, quiet, clean” at mealtime (Figures 4, 5) and “quick, quiet, on time” at bedtime (Figures 6, 7), lacking mutual communication and spiritual comfort between family members. In the classroom, teachers can guide students to analyze problems comprehensively, and attempt to pay attention to each student. In boarding life, teachers can also take the initiative to care about students, pay attention to cultivating students' ability to control and regulate emotions, and set a good emotional example for students. However, the teachers' emotional value identification education does not fit well with the specific situation of family education. Meanwhile, they ignore the foundational function of family education's emotional value identification on the physical and mental development of students. In other words, teachers should not only have good emotional qualities, but also deliver emotional energy actively in the process of educating pupils. For example, under the guidance of teachers, students realize the psychological identity of the collective concept through empathy education, so as to achieve the purpose of loving the class collective. However, subject to the constraints of professional identity, teachers may not be able to provide boarding students with the same family emotional value compared with their parents.

#### 2.1.3. Transfer of Space-Time in family education

The terrain of Nujiang Lisu Autonomous Prefecture is high in the north and low in the south. The long and narrow mountain canyon landform makes travel extremely inconvenient. To improve the enrollment rate and ensure the smooth development of compulsory education, in March 2011, all school sites under the jurisdiction of Stone Moon Township were merged leading to the formation of a centralized school running system.

The birth of boarding primary school has shifted family education from home to school. Family is the first school of all children. Originally, parents had plenty of time to educate their children at home after school every day. The places of education can be homes, fields, and villages. The family education of primary school students was entirely conducted in the family domain before the centralized school running system. The main body of family education presents diversity. Lisu people implement the monogamous marriage system and have practiced cohabitation for generations. The patriarch has the most prestige. Therefore, the subjects of children's family education are generally parents, grandparents, patriarchs, and other elders. After the children begin to live in boarding schools, subject to the needs of local economic development, many young parents choose to go out to work. The main body of family education for children gradually changed to grandparents. However, some grandparents are too old to discipline their grandchildren. The emergence of boarding schools has gradually weakened the strength of the main body of family education.

**Figure 8:**
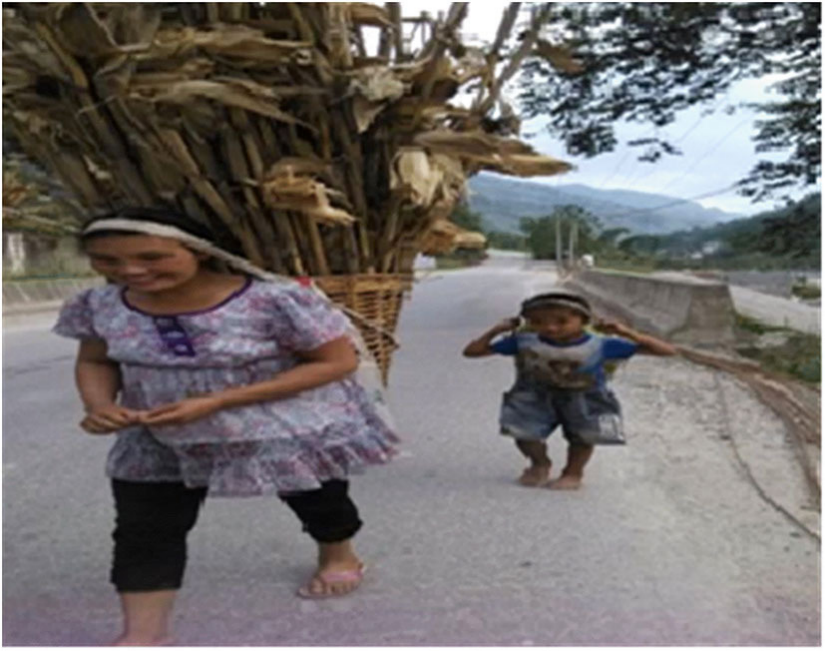
Mother and son returning after cutting wood.

**Figure 9:**
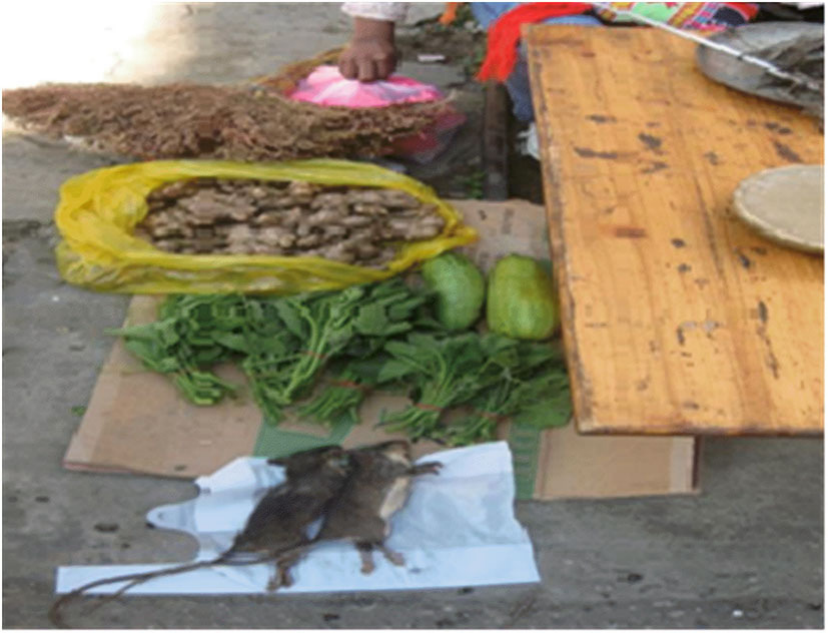
Edible mountain squirrels.

The forms of family education consist of folk customs, including traditional culture education of Lisu, labor education, ethics education, simple science, culture education, and so on. The main festival of Lisu is the “Kuoshi Festival” from the 5th day of the 12th lunar month to the first day of the first lunar month of the following year. During the festival, parents mainly lead their children to worship ancestors, exorcize ghosts and pray for blessings. “Climbing the Knife Hill” and “Crossing in the Sea of Fire” are the most exciting programs of “Kuoshi Festival.” Lisu people are fond of singing and dancing. Therefore, their children have learned various folk songs, dances, and proverbs since birth under the influence of them. Lisu parents pay more attention to training their children's living and working skills. Around the age of 5, parents begin to teach their children to go up the mountain to cut firewood (Figure 8), do laundry and cook, and take care of their younger siblings. Fathers teach their sons hunting techniques, such as catching mountain rats by smoking (Figure 9) and identifying beasts by their footprints. Mothers teach their daughters how to spin and weave. They also take their children to the mountains to pick wild fruits and identify whether they are poisonous or not. The ethical and moral education of Lisu is mainly reflected in teaching children to respect gods, respect the elders and cherish the youth, and be diligent and thrifty. In life, parents also teach their children basic scientific and cultural knowledge such as numeracy and literacy. After their children began to live in boarding schools, families handed over most of their education rights and duties to schools, and the family education of Stone Moon Primary School students became extremely limited.

### 2.2. Theoretical basis

The causes of the lack of family education in boarding primary schools in ethnic areas from the perspective of Overlapping Spheres of Influence were analyzed.

In the 1980s, Joyce L. Epstein, professor of Johns Hopkins School of Education and founder of the National Network of Partnership Schools, according to the conclusions of the *Coleman Report* “whatever the family background is, family for children's academic achievement plays a most important role “raised the question “if families are so important to children's education, how can schools involve families to promote students' success?”. When studying this problem, Professor Epstein put forward the theory of Overlapping Spheres of Influence.

**Figure 10:**
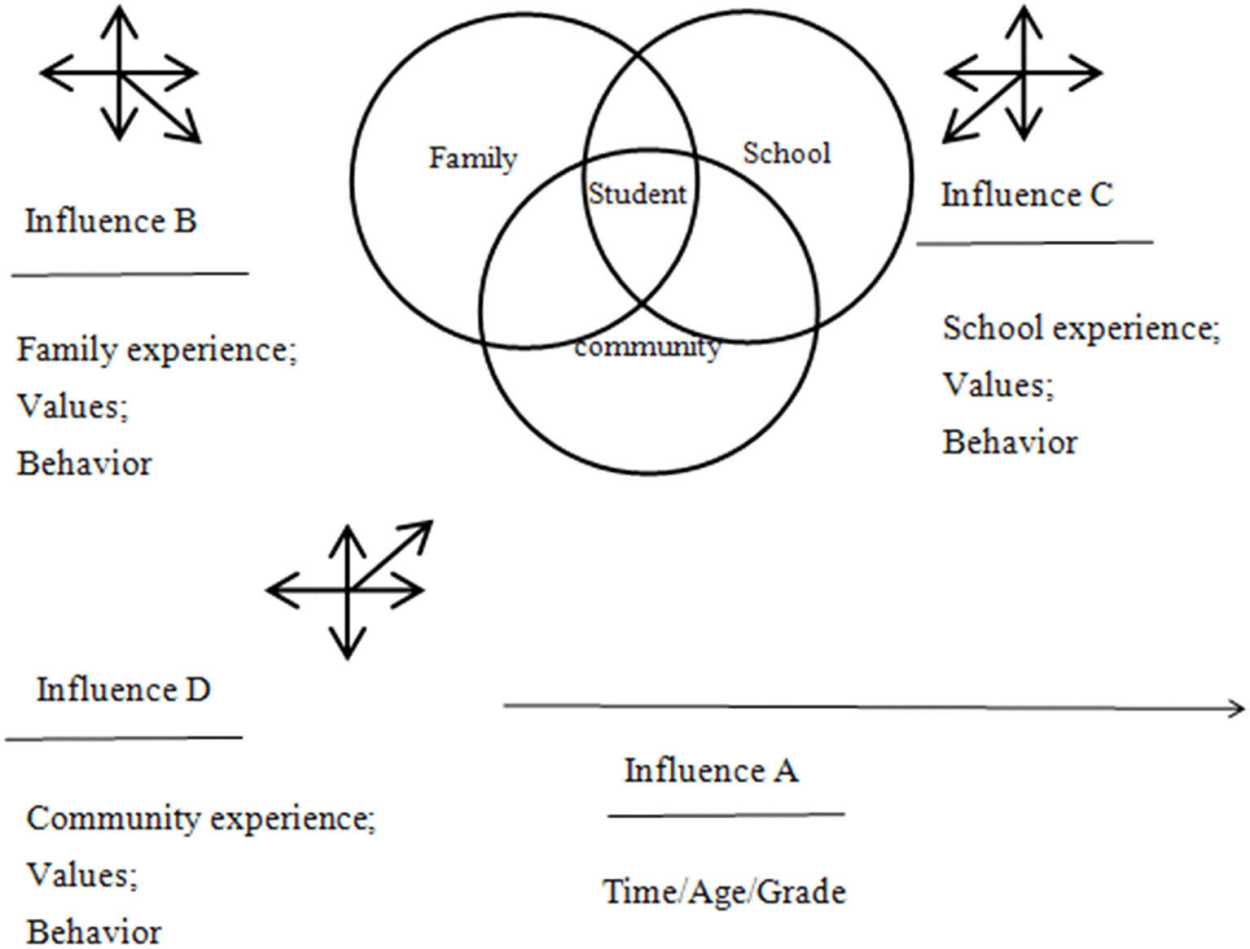
The external model of Overlapping Spheres of Influence theory.

**Figure 11:**
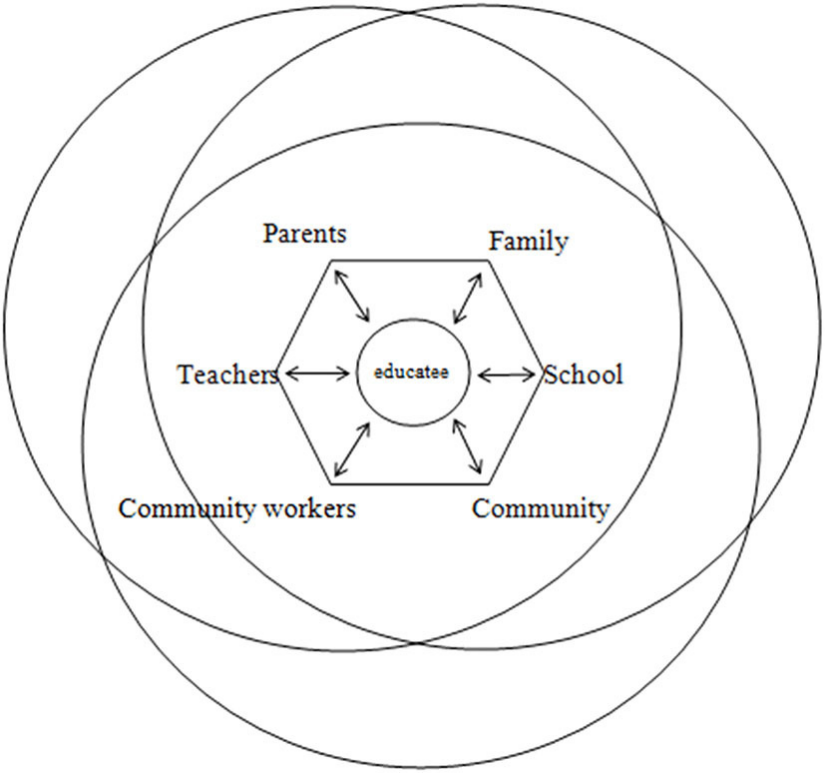
The internal model of Overlapping Spheres of Influence theory.

Overlapping Spheres of Influence theory holds that the conditions and relationships among family, school, and community all have overlapping effects on students' growth. The theory constructs student-centered external (Figure 10) and internal models (Figure 11), emphasizes the leading role of the school, and proposes six types of practical frameworks (Table 1, Epstein, 1998) to guide schools, families, and communities in forming consistent behaviors, experiences, and values. On that basis, it establishes family-like schools, school-like families, school-like communities, and family-like communities (Epstein, 2009), improves the management and teaching efficiency of schools, enhances parents' ability to nurture their children, strengthens the relationship among families, schools and communities, and upgrades the educational aspirations of students and promotes their success in schooling (Zhang et al., 2019).

**Table 1:** Types of “family-school-community” cooperation.

**Number**	**Type name**
Type 1	Parenting
Type 2	Communicating
Type 3	Volunteering
Type 4	Learning at home
Type 5	Decision-making
Type 6	Collaborating with community

The reason that the Overlapping Spheres of Influence theory can explain the causes of the lack of family education in boarding primary schools in ethnic areas is that the origin of the two lies in the interactive process of education. Educational problems generate support demands, which give birth to helping forces, which in turn produce overlapping influence.

In addition, the focus of the two is how to cooperate with each other in order to better promote the development of students and to provide a sense of life filled with explanations for the complex and dynamic educational practice. When children are separated from their parents and sent to boarding schools, the family environment, the local language, and the family culture will be weakened or even disappear.

According to the external model of Overlapping Spheres of Influence theory, it can be concluded that there is an “overlapping and separation” relationship between “family, school, and community,” and the subjects of them can cooperate or operate independently. The experience, values, and behaviors of the “home-school- community” will have an impact on students. When students come to boarding school, they will lose their family environment. At the same time, “family” will be absent from the overlapping influence on students, and “family education” will lose its role.

Therefore, boarding schools should pay attention to the construction of family life situations to create a family-like environment. According to the internal model of the Overlapping Spheres of Influence theory, it is concluded that schools play a leading role in education, because only schools are institutionalized institutions in which education exerts influence. Students are the center of “overlapping influence” and the subject of education.

Hence, boarding schools should be noted that the continuity of educational language, the consistency, and the systematicness of educational management mode are on the basis of respecting students' dominant position in learning. According to the Overlapping Spheres of Influence theory, six types of “family-school-community cooperation” are concluded: participating in decision-making, community cooperation, voluntary service, studying at home, being good parents, and communicating with each other. A practical mode for schools to assist families and communities to participate in students' growth education was born.

In the boarding school, the creation of “family culture” inherited by “family tradition, family discipline, and family construction” and “community culture” formed by “environment, behavior, system, spiritual culture” is necessary to achieve the “family, school and community” three-way linkage of student growth education practice mode. To sum up, the lack of family environment, home language, and family culture in boarding schools is referred to as the lack of family education in boarding schools in this study.

### 2.3. Research hypothesis

**Figure 12:**
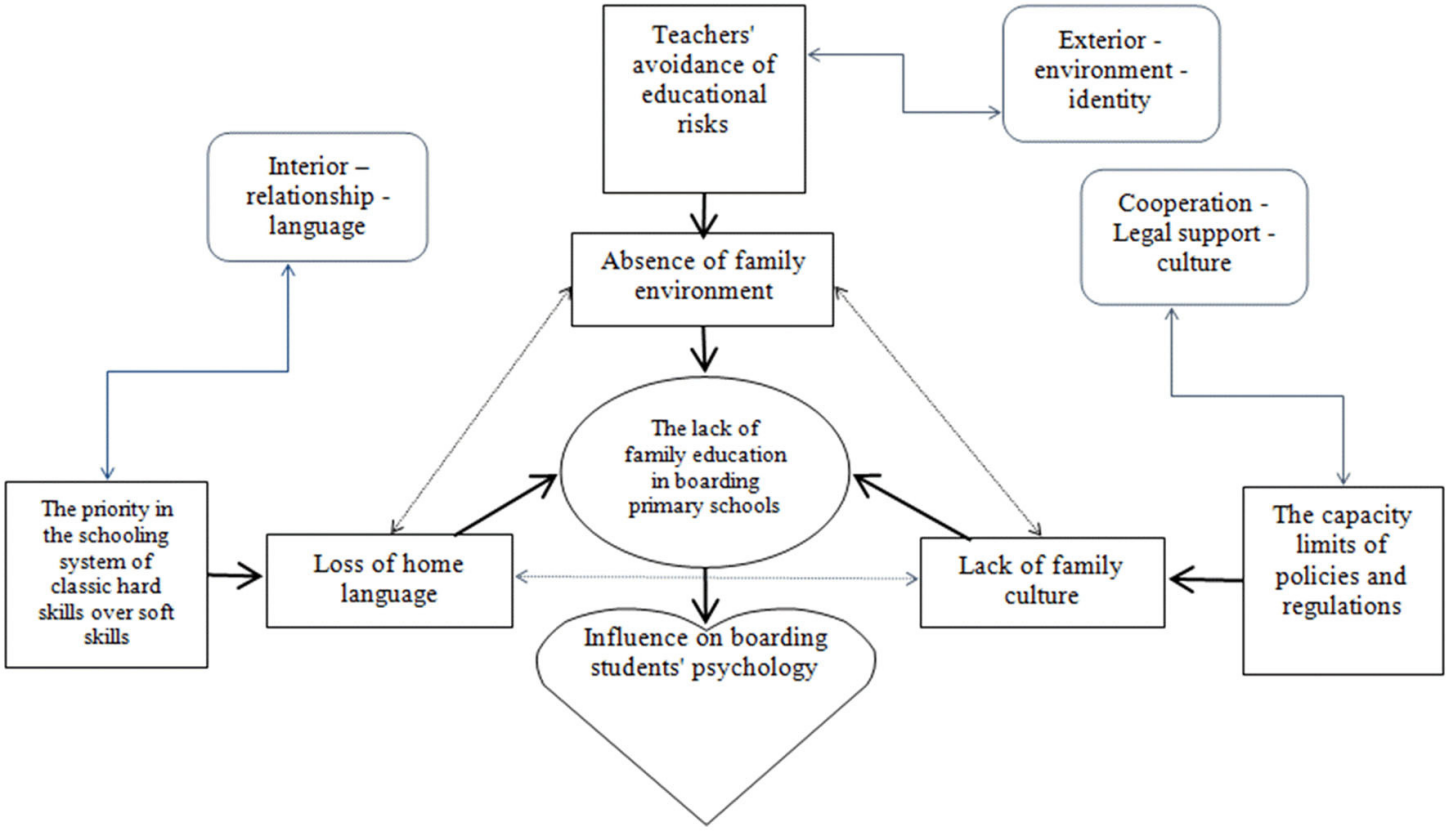
The theoretical analysis framework of the lack of family education in boarding primary school.

If boarding primary schools in minority areas neglect the construction of family life situation, the collapse of language understanding of boarding pupils and the creation of family culture, and the situation of lack of family education will be aggravated. According to the Overlapping Spheres of Influence theory, it is hypothesized that there are three main factors influencing the lack of family education in boarding primary schools in ethnic areas. First, teachers' avoidance of educational risks; second, the priority in the schooling system of classic hard skills over soft skills; and third, the capacity limits of policies and regulations. The following is the theoretical analysis framework of the lack of family education in boarding primary schools (Figure 12).

#### 2.3.1. Exterior–Environment–Identity: Teachers' avoidance of educational risks

The risks of education take many forms. For example, the teachers' input does not match the students' output, the students' academic failure could be attributed to the teacher, and so on. There are always many risks in education because education is not the filling of a bucket, but the lighting of a flame. There are always many risks because education is not an interaction between robots, but an encounter between people. Risks are inherent in education because students should not be seen as objects to be molded and disciplined but as subjects of initiating action and assuming responsibility (Biesta, 2013).

To achieve the teaching goal as best as possible, the teachers of Stone Moon Primary School are working hard to complete the teaching tasks of each course. Teachers are keen on targeted education to get pupils to do well in exams. Teachers can also predict the final teaching effect according to the syllabus, their own classroom teaching, and students' consolidation practices. Evidently, this form of teaching is relatively easy to operate and safe. However, the object of education is a living individual. In addition to their identity as students, the children also have other specific identities such as children or human beings. The pupils of Stone Moon Primary School leave home for boarding school, and their identity as children and human beings continues. Nevertheless, teachers treat students as objects of instruction and thus may lose sight of their identity as children. In short, in order to complete the teaching task, teachers neglect or even deliberately avoid the creation of family-oriented education situations, resulting in the complete isolation of boarding students from their home surroundings.

#### 2.3.2. Interior–Relationship–Language: Priority in the schooling system of classic hard skills over soft skills

Boarding schools were established to guarantee students equal access to education. The main responsibility of school education is to cultivate individuals who can promote social development and personal growth. In this process, the schools make use of various superior resources in accordance with the law to achieve their educational goals. Under the mainstream impact of school education, family education has been marginalized. Education should ultimately make education itself unnecessary—no teacher wants his students to be permanent students—which means that education must have an orientation for the educated to obtain freedom and independence. The curriculum of Stone Moon Primary School is set up according to the scientific standard of the curriculum plan, but it neglects a holistic approach and fails to make up for family-oriented educational content and atmosphere. In other words, boarding schools pay more attention to hard skills such as students' academic performance and tend to neglect soft skills such as caring for students. Pupils are required to communicate in Mandarin at school. It is easy to finish for students in grade five or six, but for students below grade three, especially for novices, they will become silent or inferior without the assistance of local language or home language. Some parents try to communicate with their children in Mandarin at home to help them adapt to the language environment of boarding schools. The language that compulsory boarding school education provides for minority children is often associated with language shift and language loss. Later, it is not just peculiar to this particular school but a more general problem. In the final analysis, school education as an institutionalized subject of rights has been overemphasized, but the limitations of its educational function confirm the insurmountable gap between school education and family education.

#### 2.3.3. Cooperation–Legal Support–Culture: Capacity limits of policies and regulations

Since the beginning of the 21st century, many laws and regulations have examined family education in boarding schools to varying degrees in China. For example, *The State* Council General Office on Comprehensively Strengthening Rural Primary Schools and the Construction of Rural Boarding School Guidance (2018) proposed an educational model “to close the family-school connection, improve the system of the family visit, give full play to the role as the parent committees and parent schools, promote the improvement of the quality of family education, and form a joint force of family and school education.” The 14th Five-Year Plan for National Economic and Social Development of the People's Republic of China and the Outline of 2035 Vision Goals (2021) proposed school education measures to “improve the conditions of small-scale rural schools and boarding schools in towns and townships, improve the quality and ability of rural teachers, and improve the care system for left-behind children”. However, these policies and requirements on boarding schools only elaborated the most basic principled norms that boarding school teachers should follow in family-school cooperation or teaching, and did not clearly define the compensatory family education responsibility of boarding schools. This kind of tendentious guidance with broad meaning is difficult to convert into operational implementation rules. The capacity limit of policies and regulations exacerbates the lack of family education in boarding schools.

## 3. Methods

According to the research hypothesis of the influencing factors of the lack of family education in boarding primary schools in ethnic areas, the following empirical research design was carried out.

### 3.1. Sample

The empirical part of this study adopted a purposive sampling method to conduct semi-structured interviews with 16 teachers at Stone Moon Primary School in Nujiang Lisu Autonomous Prefecture, Yunnan Province, China. A questionnaire survey was conducted among 175 students in Stone Moon Primary School, 175 self-designed questionnaires were distributed, and 169 valid questionnaires were collected, with an effective rate of 97%. In Stone Moon Township, 120 parents were selected for the questionnaire survey, 120 self-designed questionnaires were distributed, and 115 valid questionnaires were collected, with an effective rate of 96%.

### 3.2. Data collection

Based on the three dimensions of the research hypothesis on the influencing factors of the lack of family education in boarding primary schools in ethnic areas, two sub-questionnaires were compiled. Subquestionnaire one was “Indicators of the Lack of Family Education in Boarding Primary Schools”. This index was divided into nine items, which was used to evaluate the degree of lack of family education in boarding schools reflected by different subjects from the aspects of environment, identity, relationship, language, law, culture, and psychology. The second questionnaire was “Influencing Factors of Lack of Family Education in Boarding Primary Schools.” This index included three dimensions: “teachers' avoidance of educational risks”, “the priority in the schooling system of classic hard skills over soft skills”, and “the capacity limit of policies and regulations”. The questionnaire adopted the Likert 5-grade scoring method, with 1–5 points representing “very disapproving” to “very approving” in turn. The higher the score, the higher the degree of approval. Cronbach's α reliability coefficient method and KMO validity coefficient method were used to test the reliability and validity of the questionnaire.

The variables selected in this study included three basic types: dependent variable, independent variable, and control variable (Table 2). The dependent variable of this study was “the lack of family education.” The independent variables were “teachers' avoidance of educational risks”, “the priority in the schooling system of classic hard skills over soft skills”, and “the capacity limit of policies and regulations”. Control variables included family and individual levels. At the family level, the control variables selected included household income, annual education expenditure, family type, and loan. The control variables selected at the individual level included sex, ethnic belonging, age, grade of students, and degree of parents.

**Table 2:** Variable declaration.

**Variable types**	**Variable names**	**Variable instructions**	**Variables identify**
Dependent variable	The lack of family education in boarding primary schools.	/	LFE
Dependent variables	Teachers avoid educational risks;	Environment—Identity—psychological health;	TAR
	The priority in the schooling system of classic hard skills over soft skills	Relationship—language—psychological health;	HSP
	Capacity Limits of Policies and Regulations.	Law—culture—psychological health.	CLP
Control variables	Annual family income	CNY(W)	Income
	Annual family expenditure on education	CNY(W)	Expenditure
	Household type	1. Nuclear family (parents and children living together) 2. Intergenerational family (parents, grandparents, children living together) 3. Single-parent or reorganized family 4. Family with left-behind children	Type
	Debt problem	Y = 1;N = 0	Loan
	Sex	Y = 1;N = 0	Sex
	Age	Age at the time of interview	Age
	Grade (Student)	Grade at the time of interview	Grade
	Ethnic belonging	Ethnic minorities = 1; Han = 0	Ethnic
	Degree (Parents)	1. Graduate degree 2. College degree 3. Secondary school education 4. Primary school education or below	Degree

Based on the above analysis, the estimation equation of the lack of family education in boarding primary schools was formulated, and the correlation analysis and regression analysis were conducted. The general estimation equation in brief form is as follows:


$$\begin{array}{l} {\text{yLFE}}_{i}=\beta_{0}+\beta_{1}{\left(\text{TAR}+\text{HSP}+\text{CLP}\right)}_{i}+\beta_{2}{\text{control}}_{i}+\beta_{3}\mu_{i} \end{array}$$

*yLFE*_*i*_ as the dependent variable, represents “the lack of family education in boarding primary schools.” β_0_ indicates intercept. β_1_(TAR+HSP+CLP)_*i*_ as an independent variable includes “teachers' avoidance of educational risks,” “the priority in the schooling system of classic hard skills over soft skills,” and “the capacity limit of policies and regulations.” β_2_control_*i*_ represents the control variable. β_3_μ_*i*_ denotes the random error term.

### 3.3. Data analysis

#### 3.3.1. Correlation analysis

The correlation analysis was made among “teachers' avoidance of educational risks”, “the priority in the schooling system of classic hard skills over soft skills”, “the capacity limit of policies and regulations”, and “the lack of family education”. The results are shown in Table 3. There are statistically significant pairwise correlations among “teachers' avoidance of educational risks”, “the priority in the schooling system of classic hard skills over soft skills”, “the capacity limit of policies and regulations”, and “the lack of family education”. The correlation coefficient between “teachers' avoidance of educational risks” and “the lack of family education” is 0.660, *p* < 0.01. The correlation coefficient between “the priority in the schooling system of classic hard skills over soft skills” and “the lack of family education” is 0.598, *p* < 0.01 The correlation coefficient between “the capacity limit of policies and regulations” and “the lack of family education” is 0.678, *p* < 0.01. The results indicate that “teachers' avoidance of educational risks”, “the priority in the schooling system of classic hard skills over soft skills”, and “the capacity limit of policies and regulations” are all related to the degree of “the lack of family education”, and shows significant positive correlations.

**Table 3:** Correlation analysis of TAR, HSP, CLP, and LFE.

**Factors**	**1**	**2**	**3**	**4**
1. TAR	1			
2. HSP	0.511^**^	1		
3. CLP	0.629^**^	0.652^**^	1	
4. LFE	0.660^**^	0.598^**^	0.678^**^	1

** p < 0.01 (Two-tailed).

#### 3.3.2. Regression analysis

Taking “teachers' avoidance of educational risks”, “the priority in the schooling system of classic hard skills over soft skills”, and “the capacity limit of policies and regulations” as independent variables, and “the lack of family education” as dependent variables, stepwise regression analysis was performed (Table 4). Three factors gradually enter the model. The significance of the three models is shown as p<0.001, which indicates that the independent variables in the three models can significantly predict “the lack of family education in boarding primary schools”. In terms of collinearity, the tolerance of each factor is >0.1, and the VIF value is <10, indicating that the multiple collinearities among the constants are weak, and the result of the linear equation is valid. In model 3, “teachers' avoidance of educational risks”, “the priority in the schooling system of classic hard skills over soft skills”, and “the capacity limit of policies and regulations” are the independent variables, while “the lack of family education” is the dependent variable. The adjusted R^2^ is 0.607, which means that in the regression equation, 60.7% of the degree of “the lack of family education” can be predicted by the three factors above. With further analysis of the independent variables in the model, the coefficient estimation results show that “teachers' avoidance of educational risks” has the best explanatory power, Beta coefficient is 0.349, indicating that the stronger the risk aversion of teachers is, the higher the degree of family education deficiency in boarding primary schools is. Second, the Beta coefficients of “the capacity limit of policies and regulations” and “the priority in the schooling system of classic hard skills over soft skills” are 0.310 and 0.237, indicating that “the capacity limit of policies and regulations” and “the priority in the schooling system of classic hard skills over soft skills” can predict the degree of family education deficiency in boarding primary schools, and have statistical significance.

**Table 4:** Regression analysis of TAR, HSP, and CLP on LFE.

		**Unstandardized Coefficients**		**Standardized Coefficients**						
**Model**		**B**	**Standard Error**	**Beta**	**t**	***p***	**F**	***p***	**R ^2^**	**ΔR ^2^**
1	(Constants)	7.080	0.642		11.024	0.000	440.92	0.000	0.492	0.490
	CLP	0.394	0.019	0.701	20.998	0.000				
2	(Constants)	4.674	0.632		7.391	0.000	315.77	0.000	0.581	0.579
	CLP	0.249	0.023	0.443	11.048	0.000				
	TAR	0.200	0.020	0.396	9.869	0.000				
3	(Constants)	3.020	0.676		4.465	0.000	236.098	0.000	0.609	0.607
	CLP	0.174	0.025	0.310	6.870	0.000				
	TAR	0.177	0.020	0.349	8.801	0.000				
	HSP	0.160	0.028	0.237	5.720	0.000				

Dependent variable: LFE.

## 4. Results

The correlation analysis showed that “teachers' avoidance of educational risks”, “the priority in the schooling system of classic hard skills over soft skills”, and “the capacity limit of policies and regulations” had a significant positive correlation with the degree of “the lack of family education”.

In the regression analysis, the adjusted R^2^ was 0.607, indicating that the proportion of “lack of family education “explained by the three factors of “teachers' avoidance of educational risks”, “the priority in the schooling system of classic hard skills over soft skills”, and “the capacity limit of policies and regulations” was 60.7% through the regression relationship. Intensifying of “teachers' avoidance of educational risks”, “the priority in the schooling system of classic hard skills over soft skill”, and “the capacity limit of policies and regulations” would all lead to the aggravation of the lack of family education in boarding primary schools.

These empirical results verified the research hypothesis.

## 5. Discussion

### 5.1. Possible solutions

To solve the problem of family education deficiency in boarding primary schools in ethnic areas, we need to pay equal attention to “clarifying responsibilities” and “empowerment” (Table 5). “Clarifying responsibilities” means strengthening legal protection and implementing the rights and responsibilities of all parties in education by establishing a standard system and accountability system for boarding primary school teachers and parents in ethnic areas. However, it should be noted that though boarding schools are established to facilitate the education of students, and the effectiveness of boarding schools in the middle school stage in ethnic areas can be proved, the boarding policy for younger children in primary school is still needed to be rethought in view of its greater impact on them. In other words, governments have to make education available, accessible, acceptable, and adaptable. Educators should endeavor to meet all of the requirements of rights-based education, not only the availability and accessibility of education which national governments often focus on but also the two aspects of rights-based education that are often overlooked, acceptability of education and adaptability to local stakeholders' perspectives (Tomaševski, 2001). “Empowerment” means strengthening professional guarantee, expanding the professional autonomy of boarding primary school teachers in ethnic areas, combining the boarding nature of schools, students' physical and mental development rules, characteristics of ethnic areas, independently researching and developing compensatory courses for family education functions, and strengthening the compensatory management for family education functions in such schools. Educators must redefine their roles within the classroom, the community, and the broader society so that these role definitions result in interactions that empower rather than impede the development of students. Meanwhile, they must attempt to persuade colleagues and decision makers, such as school boards and the public that elects them, of the importance of resetting institutional goals so that the schools transform society by empowering minority students rather than reflecting society by disabling them (Cummins, 1986). The compensation for the lack of family education in boarding schools needs the support of all sectors of society. It is prudent to consider educational support that can assist boarders in the transition and then through school (Martin et al., 2021). Multidimensional intervention can be effectively administered to promote the resilience of boarding students.

**Table 5:** Solutions to the lacking of family education in boarding primary schools in ethnic areas.

**A. Clarify responsibilities, and legal guarantee**	**B. Empowerment, and professional guarantee**
Clarify the responsibilities of boarding primary school teachers and parents in ethnic areas with the will of law.	Establish a functional compensatory system for family education in boarding primary schools in ethnic areas.
Strengthen the accountability of shirking responsibility and cross-border education.	Create a family-oriented living environment for boarding primary schools in ethnic areas.
Develop an operational differential job questionnaire.	Create a life-oriented education circumstance for boarding primary schools in ethnic areas.
Restore the day school system for pupils in grades 1-3.	Provide time for pupils under grade 3 to transition from mother tongue and improve their literacy and mainstream language skills.

#### 5.1.1. Legal guarantee: Establish the responsibility and accountability standard system of teachers and parents in boarding primary schools in ethnic areas

Through the top-level design of the legislation, the responsibilities of teachers and parents of boarding primary schools in ethnic areas are demarcated, the construction of the accountability system is strengthened, and the responsibilities of teachers and parents are urged to be implemented.

First, the state should improve the educational responsibility legislation of teachers and parents in boarding primary schools in ethnic areas, establish a clear list of their responsibilities and rights, and delimit the scope of rights and responsibilities of teachers and parents as well as their co-parenting responsibilities. Constructing reasonable and effective responsibility standards for boarding primary school teachers and parents in ethnic areas, and structuring responsibility standards in these areas will also be helpful in this regard. Furthermore, the rights and obligations of teachers and parents are based on respect for students' education rights. With the democratization and legal institutionalization of Chinese society, teachers' and parents' awareness of children's rights, and especially children's education rights will eventually affect every family and grow into an accepted way of life (Zhu, 1999).

Second, we should strengthen responsibility shirking and accountability of cross-border education. For teachers and parents who shirk their responsibilities and go beyond the boundaries of education, the accountability organization shall take corresponding criticism and punishment in accordance with the standard system of responsibility for boarding primary school teachers in ethnic areas, the parent responsibility standard system for boarding primary schools in ethnic areas, the accountability system for boarding primary school teachers in ethnic areas, and the parent accountability system for boarding primary schools in ethnic areas, and order them to make corrections within a time limit.

Third, according to the actual situation, a different and operational job questionnaire for teachers and parents of boarding primary schools in ethnic areas should be developed. The workload of teachers in different subjects and different types of guardians (parents, grandparents, and other guardians) should be accurately measured to ensure the rationality of the workload and working hours of teachers and parents.

Fourth, we recommend that primary schools in ethnic minority areas restore the day school system for students in grades 1–3 according to the actual situation. Because these students are too young to bear the irreversible damage caused to them by unfamiliar living circumstances and heavy learning burden. We should consider whether it is right to trade children's broken hearts for equal educational opportunities. Family experiences are linked to children's successful adjustment to the demands of school. Sharing activities and games with parents can improve children's adaptability to school (Li and Wu, 2009). Mother monitoring, mother-child communication, mother-child activity, and father-child activity can lead to more favorable child profiles (Lv et al., 2019). Therefore, students in lower grades need to return home after school every day to feel the care, companionship, and education of their parents.

#### 5.1.2. Professional guarantee: The compensatory management of family education function in boarding primary schools in ethnic areas needs to be strengthened

Boarding primary schools in ethnic areas should actively expand their school functions, change the concept of examination-oriented education and extend the time and space of education. First, teachers should be empowered and encouraged to provide comprehensive educational care for boarding students. Second, it is necessary to create a family-oriented living environment and life-oriented teaching situations. Furthermore, developing family function compensation courses independently to compensate for the lack of family education in boarding primary schools is another makeshift. The knowledge, culture, and language of ethnic minorities should be taught as a part of the school-based curriculum (Bahry, 2012), and the schools should at least provide the home language and family culture environment after class.

Primarily, boarding primary schools in ethnic areas should establish a compensatory system of family education function and multiple steps can be taken in this regard. First, a leading group of “family education function compensation” should be established with the principal as the leader, and the vice principal and grade director as members. The group establishes an office to be responsible for the implementation of the functional compensatory work of family education in boarding primary schools. Second, rules and regulations should be instituted for compensating the family education function of boarding primary schools. The management system should be subdivided into the management of teachers, living teachers, and students. Third, a clear file of lodging students should be established. Lodging student files should include students' physical condition, psychological condition, learning condition, communication condition, and family condition in as detailed a manner as possible.

Then, boarding primary schools in ethnic areas should create a family-oriented living and cultural environment. First, a certain proportion of teachers should act as surrogate parents for students, so that each of these students can fully feel the care of their parents. In a transitional model of bilingual education, the boarding students in Grades 1–3 are allowed to use their home language so that they can remain in the home environment until they are more mature. Then, bilingual instruction can be considered so that they can develop language proficiency and content knowledge in their home language and majority language and build additive bilingualism and multiculturalism (May, 2016). Second, the school should help students create a happy living atmosphere in the dormitories and a relaxed dining environment in the restaurants. When the Lisu festival comes around, all teachers and students should celebrate together, and encourage every student to participate in the activities in various forms (including behind-the-scenes planning). Third, students should be provided a way to “Tell You Quietly”, a form of psychological catharsis and a back-channel for communication. One technique could be to paste a mailbox next to the teacher's photo on the teacher's bulletin board. Students can share all their happy and sad feelings during their stay with their trusted teachers. Teachers should make an accurate analysis, give timely feedback and incorporate it into performance assessments.

Third, boarding primary school teachers in ethnic areas should create a life-oriented teaching situation. First, boarding primary school teachers are encouraged to develop courses to compensate for the function of family education. It is a democratic decision-making process of curriculum development based on the school (Yang and Zhou, 2002). No matter the acquisition of new knowledge or the mastery of existing knowledge, it is inseparable from the active participation of students and the activities of cognitive subjects (e.g., Yang and Zhou, 2002; Zhu, 2002). The construction of a functional compensation course of family education should focus on students' adaptability and acceptability. Teachers can combine their own teaching subjects with guidance on traffic safety, food nutrition, disease prevention, mental health, behavior shaping, Lisu culture, communication etiquette, and traditional culture. This will help construct life-oriented curriculum content so that students can feel a direct learning experience and establish good learning habits (Table 6). Second, teachers should establish reasonable expectations for students. Teachers should explore the bright points of each boarding pupil with appreciation. Teachers should devote more tolerance, hold students to a higher standard, and appreciate pupils more. This will help ensure that boarding pupils can feel the care and expectation of their parents from their teachers. Third, teachers should allow the imperfect existence of education. In the teaching process, teachers treat students as independent individuals, which should be close to how they would have been treated by parents at home. Teachers only need to actively guide students in the direction they want to develop and accept students' diversity and limits. Boarding primary school teachers should pay attention to the “dialogue” (Biesta, 2010) with students in teaching, rather than “indoctrination”, so that students can engage fully in the learning process and acquire the knowledge they yearn for.

**Table 6:** Compensatory courses of family education function in boarding primary schools in ethnic areas.

**Topics of the course**	**Objectives of the course**	**Implementation process**
Ethics and morality	Respect the old and love the young, be grateful, and have ethical and moral concepts	1. Quote historical stories to develop morality and ethics
		2. Analyze life cases and pay attention to moral cultivation
Life safety	Reverence for life, and have safety awareness	1. Treat students fairly and educate them with appreciation
		2. Care for the safety of housing and transportation
Folk culture	Get to know folk stories, traditional customs, and the Lisu characteristics	1. Read classic works and taste the Lisu culture
		2. Teach writing, songs and dances, and inherit Lisu characteristics
Labor skills	Master basic crop planting, building, hunting skills, and traditional costume-making methods	1. Carry out practical activities to impart labor skills
		2. Analyze realistic achievements and praise Lisu wisdom
Social etiquette	Be open-minded and rule-conscious	1. Demonstrate and set an example and feel constrained by rules
		2. Expand life opportunities and create an integrated atmosphere

Last but not least, we believe that boarding primary schools should provide time for pupils under grade 3 to develop their mother tongue while they begin to learn Chinese as a Second Language to prepare them for later grades where Chinese will be used as a medium of instruction. It is not only necessary for pupils in ethnic minority areas to develop their mother tongue, but also important for them to learn Putonghua to improve their literacy ability and understand Chinese culture. Hence some form of bilingual instruction based primarily on the first language would seem a good option. Do not consider whether boarding is necessary until at least the students are in the fourth grade. The true happiness and joy of elementary school students do not come from their will to overcome the fear of boarding, but from the love that teachers care for them as if they were parents. On the other hand, Professor Zhang Xiao found that a lower socioeconomic status, poorer physical health, and higher levels of household chaos were associated with lower frequencies of home-based involvement exhibited by caregivers (Zhang, 2021). Therefore, the government should strengthen intervention policies to empower parents in minority areas, improve the effectiveness of family education and guarantee the time of family education and companionship.

### 5.2. Practice implications and limitations

The birth of boarding schools promotes educational equity to some extent. If children do not study at a boarding primary school, they may not be able to enjoy better educational resources. Therefore, it is no better choice for parents to send their children to study and live in an arduous environment far away from their families. However, it will make family education unacceptable to students because of boarding. The importance of family education is self-evident. Theories of attachment (e.g., Ainsworth and Bowlby, 1991) have emphasized the influential role of parents in children's lives and it is possible that boarding reduces these important influences and may stunt personal development for younger students (also see Jack, 2020; Martin et al., 2021). Our research discusses whether the school can recreate a family-like environment to compensate for the loss of genuine family and community education in the home environment. We assumed the wisdom of boarding school for minority students without examining some of the assumptions underlying compulsory boarding school. An argument can be made for the utility of boarding schools in rural ethnic minority areas at senior secondary, and perhaps junior secondary level, but based on the effects on children the policy for elementary school-aged children needs rethinking. Therefore, the next question we need to consider may not be whether or not we should compensate family education in boarding primary schools in ethnic areas, but whether our younger children should be boarded. Moreover, this study tries to reduce the negative influence of boarding on children by creating family-oriented education, but it is unlikely that these can fully compensate for the absence of familiar people, culture, and language. It is the most tangled point in this study. The limitations in our study should be considered when interpreting conclusions, which offer some direction for future research.

## 6. Conclusion

Taking Stone Moon Primary School in Nujiang Lisu Autonomous Prefecture, Yunnan Province, China as a field point, an empirical study was conducted and the following conclusions were drawn. First, the lack of family education in boarding primary schools in ethnic areas primarily reflects the absence of family education contents, the absence of family education emotion, and the Space-Time migration of family education. Second, an analytical framework for the lack of family education in boarding primary schools from the perspective of the Overlapping Spheres of Influence theory is constructed. From the dimension of External-Environment-Identity, it is analyzed that teachers' avoidance of educational risks is related to the absence of family environment in boarding primary schools. From the dimension of Internal-Relationship-Language, it is analyzed that the priority in the schooling system of classic hard skills over soft skills is related to the lack of home language in boarding primary schools. From the dimension of Cooperation-Legal Support-Culture, it is analyzed that the capacity limit of policies and regulations is related to the lack of family culture in boarding primary schools. Third, after controlling “income”, “annual family education expenditure”, “family type”, “loan”, “sex”, “ag”, “grade of students”, “ethnic belonging”, and “degree of parents”, we find that the lack of family education in boarding primary schools is significantly affected by “teachers' avoidance of educational risks”, “the priority in the schooling system of classic hard skills over soft skills” and “the capacity limit of policies and regulations”, and the influence degree reached 60.7%. Therefore, we conclude that the lack of family education in boarding primary schools in ethnic minority areas is related to three factors: “teachers' avoidance of educational risks”, “the priority in the schooling system of classic hard skills over soft skills” and “the capacity limit of policies and regulations”, and these three factors are the main factors causing the lack of family education in boarding primary schools. Fourth, possible solutions include establishing a responsibility standard system and accountability system between teachers and parents and strengthening the compensation management of family education function in boarding primary schools in ethnic minority areas. At the very least, schools should restore the day system for students in grades 1–3 and provide them with bilingual instruction based primarily on their first language. It is recommended that after these 3 years of primary education that some form of bilingual instruction should be continued to develop strong proficiency in the Lisu language and Mandarin.

## Data availability statement

All data included in this study are available upon request by contact with the corresponding author. Requests to access the datasets should be directed to YC: ynsfchenyao@163.com.

## Ethics statement

Written informed consent was obtained from the individual(s), and minor(s)' legal guardian/next of kin, for the publication of any potentially identifiable images or data included in this article.

## Author contributions

All authors listed have made a substantial, direct, and intellectual contribution to the work and approved it for publication.

## Footnotes

^1^The sun goes with the Stone Moon in the daytime, and the moon goes with the Stone Moon at night. The Stone Moon, shrouded in mist, is magnificent. Lisu compatriots regard Stone-Moon Mountain as the birthplace.

^2^This picture shows the main gate of Stone Moon Primary School, taken by the author during the investigation of Stone Moon Primary School. The figure in the picture is the author Guo Guo.

^3^February is the winter vacation in China, so the last day of the month marks the day of returning to school after winter vacation.
